# Novel therapeutic options for the emerging infectious diseases from Romania

**DOI:** 10.1186/1471-2334-13-S1-O22

**Published:** 2013-12-16

**Authors:** Bogdan Cîrciumaru

**Affiliations:** 1 Infectious Diseases Ward, Central Universitary Emergency Military Hospital Dr Carol Davila, Bucharest, Romania; 2 National Institute for Infectious Diseases “Prof. Dr. Matei Balş”, Bucharest, Romania

## Background

The (re)emergence of certain infectious diseases in Romania, as the pathology treated in our Ward expressed it, include: the West Nile virus febrile diseases (mainly meningoencephalitis), the A (H1N1) pandemic influenza, malaria, amoebiasis, *Clostridium difficile* colitis. These infections were treated in our Ward as well, as presenting specific particularities of our specialty. As far as the emergence of resistance occurred in many conditions, as well as the compliance to therapy was low for some patients, novel therapeutic options were used in order to treat those conditions.

## Methods

The clinical-epidemiological peculiarities of the patients admitted into the Institute of Infectious Diseases (mainly those from the Military Ward) were both prospectively and retrospectively studied, and several clinical patterns as well as therapeutic resistance profiles were described. We used therapies that fitted those profiles and which used new drug categories according to the most modern international guidelines for therapy.

## Results

The patterns clinically identified were described, the resistance profiles were identified, and the novel therapeutic regimens were used for treating the selected cases, according to their clinical and laboratory particularities; all these cases had been compared to the patients treated in a “classical” fashion, the results were at least equal or even better for those treated with novel therapies, compared to the “classical” ones.

## Conclusion

(Re)emerging infectious diseases, appeared as pandemics: A (H1N1) influenza, epidemics (the West Nile meningoencephalitis, *Clostridium difficile* colitis), or sporadically imported diseases (malaria, amoebiasis), most cases were produced by multi-resistant germs. Grouping the patient evolution in clinical patterns is helpful for early focusing of the medical activities towards the complicated cases that could require highly specialized medical interventions (e.g. intensive care) as well as novel therapies. So far, those new therapeutic options were satisfactory in order to provide solutions to the treatment of the complicated cases.

